# A rare case of perineal trauma in an adolescent

**DOI:** 10.1093/jscr/rjaf638

**Published:** 2025-08-21

**Authors:** Hind El Yousfi, Ismail Benomar, Loubna Aqqaoui, Houda Oubejja, Fouad Ettayebi

**Affiliations:** Department of Pediatric Surgical Emergencies, Children's Hospital, CHU Ibn Sina, Rabat Institute, PB 6527, Lamfadel Cherkaoui Street, Rabat 10000, Rabat-Salé-Kénitra Region, Morocco; Faculty of Medicine and Pharmacy, Mohammed V University – Souissi (Université Mohammed V de Rabat), Avenue Mohammed Belarbi El Alaoui, Campus d’Al Irfane, Rabat-Salé-Kénitra Region, 10000 Rabat, Morocco; Department of Pediatric Surgical Emergencies, Children's Hospital, CHU Ibn Sina, Rabat Institute, PB 6527, Lamfadel Cherkaoui Street, Rabat 10000, Rabat-Salé-Kénitra Region, Morocco; Faculty of Medicine and Pharmacy, Mohammed V University – Souissi (Université Mohammed V de Rabat), Avenue Mohammed Belarbi El Alaoui, Campus d’Al Irfane, Rabat-Salé-Kénitra Region, 10000 Rabat, Morocco; Department of Pediatric Surgical Emergencies, Children's Hospital, CHU Ibn Sina, Rabat Institute, PB 6527, Lamfadel Cherkaoui Street, Rabat 10000, Rabat-Salé-Kénitra Region, Morocco; Faculty of Medicine and Pharmacy, Mohammed V University – Souissi (Université Mohammed V de Rabat), Avenue Mohammed Belarbi El Alaoui, Campus d’Al Irfane, Rabat-Salé-Kénitra Region, 10000 Rabat, Morocco; Department of Pediatric Surgical Emergencies, Children's Hospital, CHU Ibn Sina, Rabat Institute, PB 6527, Lamfadel Cherkaoui Street, Rabat 10000, Rabat-Salé-Kénitra Region, Morocco; Faculty of Medicine and Pharmacy, Mohammed V University – Souissi (Université Mohammed V de Rabat), Avenue Mohammed Belarbi El Alaoui, Campus d’Al Irfane, Rabat-Salé-Kénitra Region, 10000 Rabat, Morocco; Department of Pediatric Surgical Emergencies, Children's Hospital, CHU Ibn Sina, Rabat Institute, PB 6527, Lamfadel Cherkaoui Street, Rabat 10000, Rabat-Salé-Kénitra Region, Morocco; Faculty of Medicine and Pharmacy, Mohammed V University – Souissi (Université Mohammed V de Rabat), Avenue Mohammed Belarbi El Alaoui, Campus d’Al Irfane, Rabat-Salé-Kénitra Region, 10000 Rabat, Morocco

**Keywords:** perineal trauma, adolescent, pneumoperitoneum, peritoneal breach, surgical exploration

## Abstract

Perineal trauma in children is rare but constitutes a surgical emergency due to the risk of deep injuries, particularly digestive lesions. We report the case of a 14-year-old boy, with no significant medical history, admitted with a perineal wound extending toward the anal margin. Clinical examination revealed low-grade fever (38°C) and a soft but tender abdomen. Abdominal radiography showed extensive pneumoperitoneum, suggesting digestive perforation. The patient underwent urgent surgical exploration under general anesthesia, revealing a peritoneal tear that was immediately sutured. This case highlights the need for a thorough assessment—including imaging and per-operative exploration—for any suspicious perineal injury. Prognosis depends on prompt treatment to prevent early infectious complications and long-term functional sequelae.

## Introduction

Perineal trauma in children is an uncommon condition, accounting for approximately 0.2% to 8% of pediatric trauma cases [1, 2]. Although infrequent, these injuries can be severe due to the risk of early infection and long-term functional sequelae [3]. Mechanisms include falls, direct blunt trauma, or impalement injuries [4]. Due to the complex anatomy of the perineum, evaluation requires a rigorous approach—often under general anesthesia—including clinical examination, imaging, and in some cases, surgical exploration [1, 5]. In cases of deep anorectal injuries, diversion colostomy may be necessary to limit contamination and preserve continence [4, 5]. We present a case of perineal trauma in an adolescent, complicated by pneumoperitoneum, which required urgent surgical exploration and early repair.

## Case presentation

We report the case of a 14-year-old male with no significant medical or surgical history, admitted to the pediatric surgical emergency unit following a perineal injury caused by a fall onto a foam stick from his own height.

**Figure 1:**
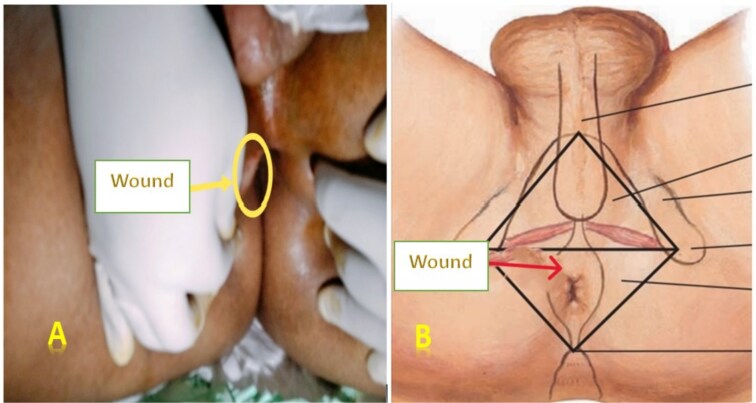
Images of the perineal wound located on the right anterolateral side. Legends: (A) Actual photograph of the perineal wound. (B) Illustrative image showing the wound location.

On admission, the patient was conscious, eupneic, and febrile (38°C), with stable hemodynamic and respiratory parameters. Abdominal examination revealed mild diffuse tenderness without guarding or rigidity. Local examination showed a clean, linear wound in the anterior perineal region, located anterior and to the right of the anus, without visible involvement of the anal margin (Fig. 1). Digital rectal examination was painless, with no bleeding or palpable injury to the anal canal.

Spontaneous micturition was noted, and urine appeared macroscopically normal, suggesting no initial urinary tract injury.

**Figure 2:**
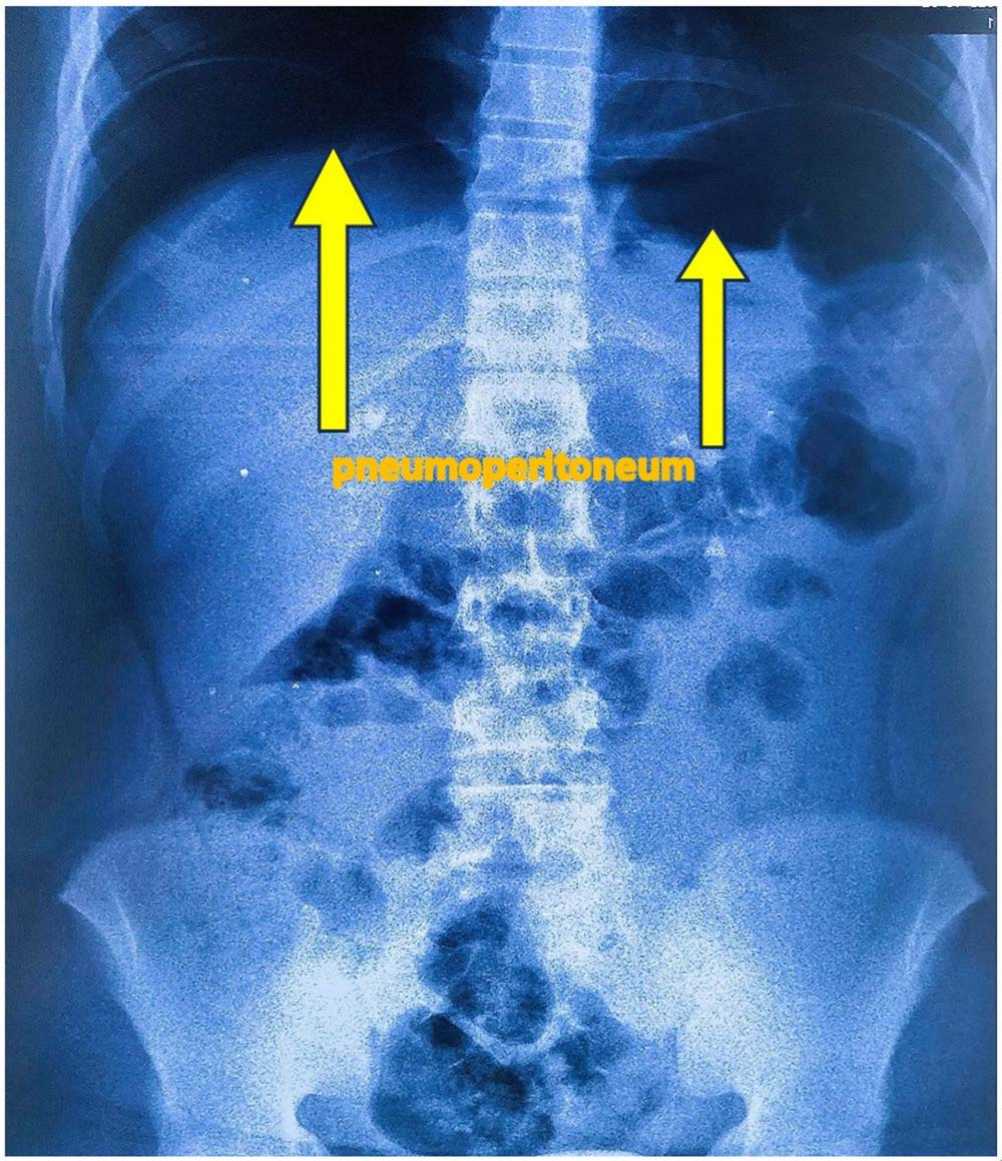
Standing abdominal plain X-ray showing large bilateral pneumoperitoneum.

**Figure 3:**
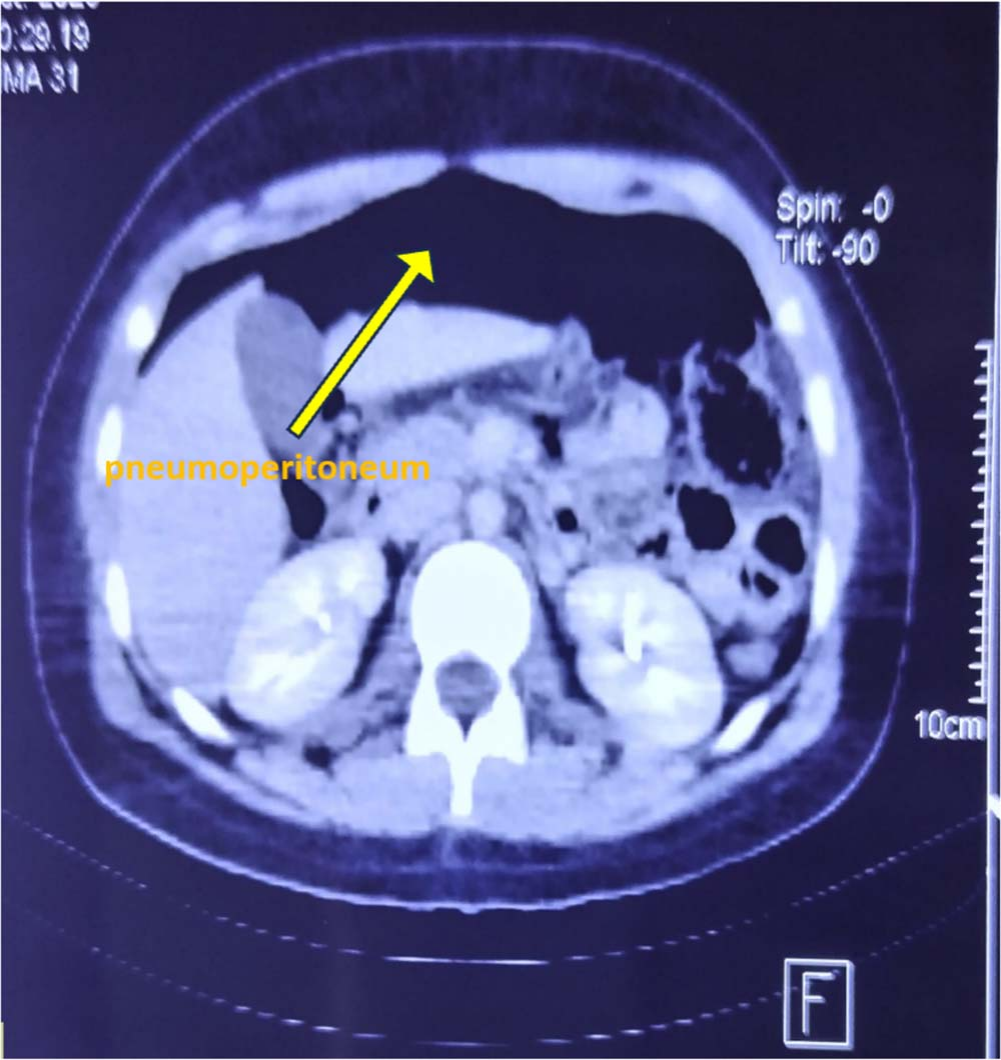
Axial CT scan of the abdomen showing extensive pneumoperitoneum.

An upright abdominal X-ray revealed massive bilateral pneumoperitoneum (Fig. 2), contrasting with the subtle abdominal signs. This clinical-radiological mismatch prompted an abdominopelvic CT scan, which confirmed the presence of abundant free intraperitoneal air, without clearly identifying the perforation site (Fig. 3).

In view of the confirmed pneumoperitoneum and absence of overt peritoneal signs, emergency surgical exploration was indicated, with strong suspicion of hollow organ perforation. Surgery was performed 12 hours after the trauma**,** following patient stabilization.

**Figure 4:**
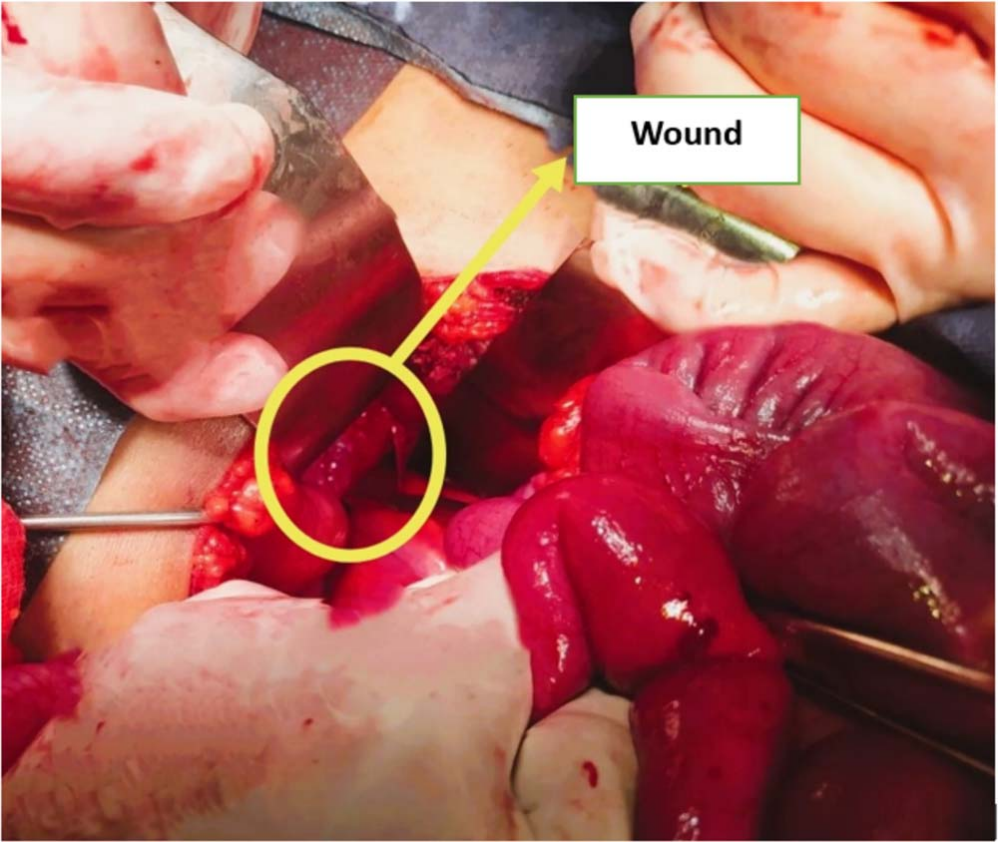
Intraoperative image showing a tear in the anterior fold of the peritoneal reflection of the pouch of Douglas.

Under general anesthesia, a transverse laparotomy (left-sided Pfannenstiel incision) was carried out. Intraoperatively, the peritoneal cavity was clean, with no fluid collection or contamination, and no rectal or sigmoid perforation. Blood clots were observed in the pouch of Douglas, and a peritoneal tear was identified on the anterior fold of the pouch, located in front of an intact rectum (Fig. 4), following a contusive trajectory toward the perineal wound.

The peritoneal breach was sutured, followed by thorough warm saline irrigation. A 14 Fr Redon drain was placed prophylactically.

Postoperative recovery was uneventful. The patient passed flatus the same day and resumed oral intake the following day. Sphincteric function remained intact, with no anal or urinary incontinence and no neurological deficit. Clinical follow-up at day 7, day 15, and one month confirmed favorable outcomes without complications.

## Discussion

Perineal trauma in children is rare, comprising 0.2% to 8% of all pediatric trauma cases [1, 2]. Although these injuries may initially appear minor, they can mask severe underlying damage involving the gastrointestinal, urinary, or reproductive systems [3]. The most common injury mechanisms include falls, direct blunt trauma, impalement, and, more rarely, sexual assault [1, 4].

In our case, a 14-year-old adolescent sustained a perineal injury after falling on a blunt object. Initial assessment revealed a clean wound without apparent anal margin involvement and no significant abdominal signs. However, massive pneumoperitoneum on X-ray and CT scan contrasted with the subtle clinical presentation, underscoring the diagnostic challenge posed by certain perineal traumas, where deep injuries may present with mild symptoms [2, 5].

The literature advocates for a systematic and thorough evaluation in such cases. In a series of 75 pediatric cases, Bakal *et al.* reported that 85% of patients required examination under general anesthesia (EUA) to accurately assess injury extent [6]. Manjunath *et al.*, in a study of 41 cases, emphasized the importance of combining imaging (X-ray, CT) with EUA to detect occult anorectal or peritoneal lesions [3].

In our case, surgical exploration identified an isolated tear in the anterior peritoneal fold of the Douglas pouch, with no rectal or sigmoid injury. Although rare, such injuries have been reported in the literature, particularly where oblique blunt force leads to localized peritoneal rupture without full-thickness bowel injury [7]. The absence of fecal contamination, pus, or digestive effusion justified a conservative surgical strategy involving simple suture of the peritoneal breach, lavage, and drainage.

A diversion colostomy was not deemed necessary, in accordance with current recommendations, which reserve this intervention for full-thickness anorectal injuries or significant contamination [3, 4, 6]. In Bakal’s series, colostomy was only performed in transmural injuries. Similarly, Palmisani *et al.* note that in low- and middle-income countries, where endoscopy is often unavailable, surgical exploration is more frequently employed [1].

Our patient’s postoperative course was favorable, with early recovery of bowel function, no infectious complications, and complete healing by day 15. These outcomes align with those reported by Sounkere-Soro *et al.*, who observed that timely management of pediatric perineal trauma results in low complication rates (9.3%) and good functional outcomes, particularly in the absence of anorectal injury [8].

Compared to published cases, our observation illustrates an atypical yet plausible scenario of isolated perineal trauma resulting in a peritoneal breach without digestive perforation. It highlights the necessity of a structured diagnostic protocol for any perineal wound in children or adolescents, regardless of initial clinical severity. Systematic imaging, EUA, and exploratory surgery in doubtful cases optimize diagnostic accuracy and allow tailored management strategies.

## Conclusion

This case illustrates how an apparently minor perineal wound can conceal a significant peritoneal injury, warranting urgent surgical intervention. Although rare, this type of lesion should be considered in any case of perineal trauma, even in the absence of overt peritoneal signs. Early diagnosis and repair are critical for ensuring favorable functional outcomes and preventing infectious complications. This case supports the importance of a multidisciplinary and protocol-driven approach in evaluating pediatric perineal injuries.

## Data Availability

No additional data are available.
